# Navigating Chinese-led economic integration: a comparative study of commercial dispute resolution and public policy exceptions in Cambodia, Pakistan and Thailand

**DOI:** 10.3389/fsoc.2026.1852024

**Published:** 2026-07-28

**Authors:** Muhammad Yaseen, Korntima Phattanasin, Shubham Pathak

**Affiliations:** 1 School of International Law, Southwest University of Political Science and Law, Chongqing, China; 2 School of Law, Center of Geosocial and Cultural Research for Sustainable Development (GSCR), Walailak University, Nakhon Si Thammarat, Thailand; 3 School of Accountancy and Finance, Center of Excellence in Sustainable Disaster Management (CESDM), Walailak University, Thai Buri, Thailand

**Keywords:** attribution asymmetry, Belt and Road Initiative (BRI), foreign direct investment (FDI), international commercial arbitration, public policy exception, sovereign shielding

## Abstract

The rapid expansion of Chinese-led economic integration under the Belt and Road Initiative (BRI) has intensified pressure on domestic dispute resolution systems across emerging Asian economies. While international commercial arbitration offers a neutral risk-management mechanism, its effectiveness relies entirely on enforcement by national courts under the 1958 New York Convention. This article investigates the escalating friction between transnational arbitral norms and host-state sovereignty through a comparative doctrinal analysis of Cambodia, Pakistan, and Thailand. Analyzing landmark disputes, the study challenges the orthodox “legal vacuum” theory, which attributes enforcement failures solely to institutional incapacity. Instead, it posits a spectrum model of sovereign shielding. Cambodia exhibits mechanical logistical bottlenecks; Pakistan demonstrates reactive judicial defensiveness stemming from sovereign trauma; and Thailand illustrates a “Capacity–Protectionism Paradox,” weaponizing sophisticated administrative law to annul high-value awards. Furthermore, the article introduces the “attribution asymmetry” dilemma, arguing that strict application of the clean hands doctrine inadvertently allows corrupt host states to evade liability while retaining infrastructural benefits, and that the sovereign shielding spectrum is predictive in nature. This research paves the way for future microlevel research to understand hybrid dispute-resolution architectures, including CICC-linked one-stop mechanisms, multi-tier dispute clauses, dispute adjudication boards, and mediated settlements under the Singapore Convention.

## 1 Introduction

The expansion of transnational development initiatives over the past two decades, most prominently the Belt and Road Initiative (BRI), has significantly reshaped global capital-flow patterns and the operational environment of international economic law (Khan et al., 2018; Dahlan M, 2020; Dahlan M. R., 2020; Dahlan, 2018). By mobilizing vast investment into infrastructure, energy, and logistics networks across the Indo-Pacific, Chinese-led economic integration has largely proceeded through a flexible, partnership-oriented governance model that relies more on non-binding memoranda of understanding than on formal multilateral treaty frameworks (Dahlan, 2020).

While this decentralized, infrastructure-driven approach has enabled rapid geographic expansion, it has also exposed a persistent structural vulnerability: uneven readiness across host-state commercial dispute resolution (CDR) systems. As cross-border mega-projects move from diplomatic conception into complex, long-term operational phases, weaknesses in domestic adjudicatory frameworks have become increasingly visible. This has intensified scholarly and policy attention to what is often described as the emerging “Legal Belt and Road” (Harkavy, 2025).

Strengthening credible, rules-based dispute resolution mechanisms has therefore become an important strategic priority for sustaining investor confidence, managing sovereign risk, and stabilizing long-horizon infrastructure cooperation (Moreira and Lin, 2025). Yet the rapid influx of foreign direct investment (FDI) frequently places substantial pressure on domestic legal systems whose institutional development may lag the pace and scale of economic integration (Harahap, 2025). In many host states, ordinary commercial litigation continues to face persistent constraints, including court congestion, limited commercial specialization, and varying degrees of political sensitivity (Opeskin, 2022).

At the same time, traditional Investor–State Dispute Settlement (ISDS) mechanisms have encountered growing sovereign pushback, driven by concerns over cost, duration, and perceived encroachment on regulatory autonomy (Teramura et al., 2024). International commercial arbitration is often positioned as a neutral alternative. However, its practical effectiveness remains critically dependent on the enforcement posture of domestic courts under the 1958 New York Convention (Born, 2021).

To interrogate the evolving friction between transnational arbitral norms and domestic legal sovereignty, this article conducts a comparative analysis of Cambodia, Pakistan, and Thailand. This article adopts a comparative socio-legal and doctrinal method to examine how public policy exceptions operate across different stages and types of legal integration under Chinese-led economic cooperation. Cambodia, Pakistan, and Thailand are selected not merely because they are BRI-linked jurisdictions, but because they represent three analytically distinct positions within the regional legal-institutional spectrum. Cambodia illustrates a transitional post-conflict legal order where arbitration reform has been formally adopted but remains constrained by weak judicial specialization and administrative capacity (West, 2018). Pakistan represents a common-law jurisdiction shaped by colonial statutory inheritance, federal complexity, and heightened sovereign sensitivity following major investor–state disputes (Ghouri, 2025). Thailand, by contrast, represents a more institutionally mature civil-law system with established arbitral institutions, yet one that deploys administrative law and public policy review to manage exposure to high-value awards (Nupueng et al., 2018).

This selection, therefore, allows the article to compare not only different national legal systems but also different modes of host-state adaptation to transnational commercial norms. The cases are not statistically representative of all BRI jurisdictions but are analytically selected case studies designed to reveal a graduated spectrum of enforcement friction. This design strengthens the central argument that enforcement outcomes cannot be explained solely by the “legal vacuum” thesis but must also be understood in terms of institutional maturity, legal culture, and strategic sovereignty behavior.

Cambodia reflects a transitional post-conflict economy facing significant capacity constraints; Pakistan represents a common-law system shaped by statutory legacy and ISDS exposure; and Thailand presents a more institutionally mature environment that nonetheless exhibits notable judicial and regulatory resistance.

The central contribution of this article is to develop a sovereign-shielding spectrum model that explains why enforcement resistance varies among host states exposed to transnational infrastructure disputes. Rather than treating enforcement failure as a simple consequence of weak institutions, the article argues that resistance evolves through the interaction among legal capacity, fiscal exposure, bureaucratic incentives, and sovereign sensitivity. At the lower end of the spectrum, Cambodia illustrates capacity-driven shielding, where enforcement difficulties arise mainly from logistical and institutional bottlenecks. At the intermediate level, Pakistan demonstrates reactive judicial shielding, where statutory legacy and sovereign trauma produce expansive judicial intervention and volatile public policy review. At the higher-capacity end, Thailand illustrates strategic doctrinal shielding, where sophisticated administrative law and public policy reasoning are mobilized to manage exposure to high-value awards. This model challenges the linear assumption that legal modernization necessarily produces stronger enforcement. Instead, it shows that enhanced legal capacity may, under certain political and institutional conditions, equip states with more refined tools of sovereign protectionism. Building on this framework, the article also develops the concept of attribution asymmetry in corruption-related disputes and argues for hybrid and multimodal dispute-resolution architectures as practical mechanisms to reduce domestic enforcement traps in Chinese-led economic integration.

## 2 Institutional design and public policy in transnational commercial disputes

The rapid expansion of transnational capital, particularly under the Belt and Road Initiative (BRI), has not only increased the volume of cross-border commercial activity but has also exposed important structural tensions within domestic dispute resolution systems. As major infrastructure and investment projects transition from political commitment to long-term implementation, they inevitably intersect with the institutional realities of host states (Khan et al., 2018). The effectiveness of commercial dispute resolution (CDR) frameworks in emerging markets must therefore be assessed in context. It is shaped not only by statutory design but also by deeper factors, including historical legal development, political economy, and administrative capacity to absorb foreign direct investment (FDI) (Harahap, 2025).

To understand the frictions that arise when transnational arbitral norms encounter domestic judiciaries in Cambodia, Pakistan, and Thailand, this article moves beyond orthodox accounts that focus primarily on “legal capacity deficits.” Instead, it situates contemporary dispute governance at the intersection of three analytical perspectives: institutional rationality, contextual resistance to legal transplantation, and the strategic legal ordering associated with the contemporary expansion of Chinese capital. Together, these lenses provide a more nuanced framework for explaining why formally pro-arbitration jurisdictions may nevertheless produce uneven enforcement outcomes.

### 2.1 Institutional rationality and the politics of investor–state friction

Conventional accounts of international commercial arbitration often portray arbitral tribunals as neutral and largely autonomous adjudicatory bodies. Yet, when examined through the lens of institutional design, arbitration reveals an embedded political economy. Transnational investors typically favor highly autonomous and enforcement-oriented mechanisms to mitigate perceived risks associated with domestic courts in host jurisdictions (Opeskin, 2022). From the investor’s perspective, arbitration offers procedural flexibility, neutrality, and insulation from local political pressures.

However, this institutional architecture contains an inherent structural dependency. Arbitral tribunals lack independent coercive authority and must ultimately rely on domestic courts for recognition and enforcement under the New York Convention (Calabrese et al., 2022; Nottage and Thanitcul, 2016). This dependence creates a predictable site of tension. Host states face competing incentives: they seek to attract foreign investment and signal legal reliability, yet they also remain sensitive to fiscal exposure, regulatory autonomy, and domestic political accountability (Poulsen, 2015).

When large arbitral awards threaten these sovereign interests, domestic legal systems may respond by recalibrating the balance between openness and control. Rather than uniformly demonstrating pro-enforcement compliance, some states maintain or develop institutional filters such as expansive judicial review pathways or restrictive approaches to arbitrability that preserve a degree of sovereign oversight (Nottage and Thanitcul, 2017). In this sense, the governance of transnational disputes can be understood as a structured form of “co-opetition,” in which states simultaneously facilitate foreign investment while retaining legal mechanisms that manage exposure to adverse outcomes (Teramura et al., 2024).

### 2.2 Contextual resistance and the limits of legal transplantation

Persistent enforcement frictions in the Global South also invite reconsideration of traditional theories of legal transplantation. Earlier comparative law scholarship suggested that adopting formal legal frameworks from advanced jurisdictions, most notably the UNCITRAL Model Law, would signal market readiness and promote institutional modernization (Watson, 1974). The implicit assumption was that statutory convergence would gradually produce functional convergence (Abala and Koh, 2025).

The comparative experience of Cambodia, Pakistan, and Thailand suggests a more complex reality. In many instances, economic integration has outpaced the development of supporting legal culture, bureaucratic practice, and judicial specialization. Contemporary scholarship increasingly recognizes that the transplantation of statutory text does not automatically reproduce the institutional conditions necessary for effective implementation (Hoang Tu Linh, 2025).

Across Southeast and South Asia, layered colonial legacies, post-conflict reconstruction, and rapid economic liberalization have produced hybrid legal orders that do not always internalize imported commercial norms in predictable ways (Chesterman, 2017). Within these environments, domestic courts rarely reject international arbitration outright. Instead, they often engage in selective adaptation. What may appear externally as inconsistency can reflect forms of contextual resistance shaped by domestic administrative law, constitutional structure, and political–business relationships (Goh and Tangkiriphimarn, 2023).

The public policy exception under Article V(2)(b) of the New York Convention plays a central role in this filtering process (Llamzon, 2014). Because the Convention leaves the content of public policy largely to national courts, it creates doctrinal space through which domestic priorities can be asserted. Expansive or context-sensitive interpretations of public policy, therefore, function not only as legal doctrine but also as instruments through which states negotiate the local impact of transnational awards. From this perspective, developing states should be understood not simply as passive recipients of transplanted arbitration regimes, but as active legal actors shaping the contours of transnational economic governance (Trzcinski and Upham, 2014).

### 2.3 Legal actors as social intermediaries in public policy review

A sociology-of-law perspective requires attention to the role of legal actors who operationalize public policy exceptions in practice (Edelman and Stryker, 2005). Public policy is not self-executing; it is constructed through interpretation by judges, government lawyers, arbitrators, corporate counsel, and bureaucratic officials who mediate between international legal norms and domestic institutional realities.

In Cambodia, the interpretive role of legal actors is constrained by institutional fragility and limited specialization (Louth, 2015). As a result, enforcement outcomes are shaped more by procedural delay and administrative capacity constraints than by doctrinal sophistication.

In Pakistan, judicial and state legal actors operate within a legal culture influenced by common-law adversarialism and heightened sensitivity to sovereign liability following major ISDS disputes (Zeeshan et al., 2024). This shapes a more defensive interpretation of public policy, particularly where state assets or fiscal exposure are involved.

In Thailand, legal actors are embedded in a strong administrative-law tradition and bureaucratic accountability structure (Sahl and Syafiq, 2025). This produces a more technically sophisticated deployment of public policy, where doctrines such as prescription periods, administrative legality review, and corruption-based invalidation are used as structured legal tools.

Across all three jurisdictions, hybrid dispute architecture is therefore not only institutional but also social, produced through the interpretive practices of legal professionals embedded in different political and administrative cultures.

### 2.4 Strategic legal ordering and the Chinese law and development (CLD) approach

The scale and distinctive institutional character of Chinese outbound investment under the BRI further complicate traditional models of dispute governance. Recent scholarship on “Inter-Asian Legalities” suggests that Chinese law-and-development practice tends to emphasize pragmatic coordination and flexible ordering rather than formal legal harmonization (Erie et al., 2025).

Unlike earlier Western law-and-development initiatives, which often sought to reshape host-state legal systems as a precondition for investment, the Chinese approach has generally been more adaptive. Chinese state actors and state-owned enterprises frequently operate with explicit awareness of institutional fragility and sovereign sensitivity in host jurisdictions (McCarthy and Un, 2017). Rather than prioritizing deep domestic legal reform, they often pursue strategic legal ordering to manage risk within existing institutional environments.

This strategy is evident in forum-selection practices that favor arbitration seats in established regional hubs such as Hong Kong and Singapore, as well as in the widespread use of multi-tier dispute-resolution clauses in infrastructure contracts (Wang et al., 2024). It is also reflected in newer institutional innovations. The China International Commercial Court (CICC), for example, represents an effort to construct a hybrid dispute-resolution ecosystem combining litigation, mediation, and arbitration within a coordinated procedural framework (Moreira and Lin, 2025).

Viewed in this light, contemporary BRI dispute governance does not point toward full legal convergence. Rather, it reflects the emergence of a plural and strategically layered transnational legal order that operates alongside and at times partially around the domestic legal systems of host states (Zhang et al., 2025). This analytical framework provides the foundation for the country-specific examination that follows.

## 3 The political economy of arbitration and institutional friction

The practical effectiveness of transplanted commercial dispute resolution mechanisms cannot be evaluated solely by reference to formal statutory design. In practice, enforcement outcomes are shaped by the surrounding political economy, historical legal development, and domestic enforcement culture (Harahap, 2025). A comparative examination of Cambodia, Pakistan, and Thailand illustrates that arbitration frameworks in emerging markets rarely operate as neutral or frictionless systems. Rather, they function as contested institutional spaces in which transnational commercial norms interact sometimes uneasily with domestic sovereign priorities (Wang et al., 2024).

Moving beyond descriptive accounts of court structures, this section examines how arbitral architectures are shaped, constrained, or recalibrated in response to large inflows of foreign direct investment (FDI) (Khan et al., 2018). Across the three jurisdictions, institutional friction manifests along a broad spectrum: from structural bottlenecks in transitional systems to legislative and judicial volatility in common-law settings, and ultimately to more sophisticated forms of regulatory retrenchment in institutionally mature environments.

### 3.1 Cambodia: post-conflict institutionalization and structural bottlenecks

Cambodia’s commercial dispute resolution landscape has developed in close parallel with its post-conflict reconstruction and sustained reliance on foreign investment (Trzcinski and Upham, 2014). As the economy has diversified into manufacturing, real estate, and infrastructure development, the scale and complexity of cross-border commercial relationships have increased accordingly (Kenh and Wei, 2025). In response, Cambodia pursued a largely top-down legal-transplantation strategy intended to signal its institutional readiness to international investors.

This effort was formalized through the enactment of the Law on Commercial Arbitration in 2006, which closely follows the UNCITRAL Model Law, and the subsequent establishment of the National Commercial Arbitration Center (NCAC) in 2013. At the level of formal legal architecture, these reforms positioned Cambodia within the mainstream framework of contemporary international arbitration (Massin, 2025).

However, the functional performance of this framework remains constrained by uneven institutional development. The creation of a modern arbitral body was not matched by the parallel establishment of a specialized commercial judiciary (McCarthy and Un, 2017). As a result, applications relating to interim relief, jurisdictional review, or award enforcement must still pass through general civil courts that often lack deep commercial expertise (Srun, 2024).

These courts continue to face resource limitations, administrative opacity, and uneven technical capacity (Lawrence, 2020). In addition, overlapping ministerial authority, particularly in land and concession disputes, can further complicate enforcement pathways (Tek, 2022). Taken together, Cambodia’s principal friction appears largely structural rather than strategic. Enforcement difficulties tend to reflect administrative and institutional constraints more than a deliberate doctrinal resistance to arbitration (Herman, 2015). This positions Cambodia at the lower-capacity end of the comparative spectrum developed in this article.

### 3.2 Pakistan: sovereign anxiety and legislative inertia

Pakistan presents a more complex and volatile configuration. Operating within a deeply entrenched common law tradition, the country’s arbitration framework remains heavily shaped by the colonial-era Arbitration Act 1940. Despite its historical importance, the statute was not designed for the demands of contemporary transnational commerce and continues to permit extensive judicial intervention at multiple stages of the arbitral process [Centre for Chinese Legal Studies (CCLS), 2024].

The rapid expansion of projects under the China–Pakistan Economic Corridor (CPEC) has placed this legacy framework under increasing strain (Khalid, 2020). Multi-layered contractual arrangements involving federal ministries, provincial authorities, and foreign contractors have raised the stakes of dispute resolution. At the same time, the procedural openness of the 1940 Act continues to invite curial involvement.

Pakistan’s institutional posture has also been shaped by significant investor–state disputes, most notably the Reko Diq arbitration. The scale of potential liability in that case generated substantial fiscal and political sensitivity within the state apparatus (Abdullah and Rasool, 2026). In this environment, domestic courts have at times adopted a defensive orientation, including issuing anti-arbitration injunctions and expansive readings of public policy (Saeed et al., 2025).

These dynamics are further complicated by Pakistan’s federal constitutional structure, in which divisions of authority over natural resources and energy projects can produce intergovernmental friction and delays (Amir and Manzoor, 2025). Although the proposed Draft Arbitration Act 2024 signals awareness of these systemic pressures and an intention to align more closely with UNCITRAL standards (Iqbal, 2024), legislative delays have left the current regime in a state of partial transition.

Pakistan, therefore, illustrates a form of doctrinal volatility generated by the interaction of statutory legacy, sovereign risk sensitivity, and institutional backlog. Its experience occupies the intermediate position in the spectrum of enforcement friction identified in this study.

### 3.3 Thailand: institutional maturity and regulatory retrenchment

Thailand presents the most theoretically revealing case within the comparative framework. As an upper-middle-income economy with a modern Arbitration Act (2002) broadly aligned with the UNCITRAL Model Law and well-established institutions such as the Thai Arbitration Institute (TAI) and the Thailand Arbitration Center (THAC), Thailand demonstrates a high level of formal arbitration capacity (Kaewparadai, 2019; Bunnag, 2011).

Notwithstanding this institutional maturity, Thailand has developed a more calibrated and legally sophisticated approach to managing arbitral exposure. Following several high-profile disputes involving state agencies, notably the Hopewell and Walter Bau cases, domestic political sensitivity toward large arbitral liabilities increased markedly (Khoman et al., 2024).

In response, the Thai Cabinet adopted resolutions in 2004 and 2009 requiring government entities to obtain special approval before agreeing to arbitration clauses in administrative contracts (Sucharitkul, 2014). Although these measures do not amount to a formal prohibition, they introduce additional procedural and political filters into the state’s engagement with arbitration and signal a more cautious institutional posture.

At the bureaucratic level, the Tort Liability of Officials Act 1996 and related accountability mechanisms have also shaped decision-making incentives. Thai officials may face personal liability exposure in cases involving alleged mismanagement of public funds (Henderson, 2009). This legal environment can encourage risk-averse behavior and a preference for dispute fora, particularly the Administrative Courts, where the state retains greater procedural familiarity and control.

Thailand, therefore, illustrates how a legally sophisticated system may nonetheless generate meaningful enforcement friction. The key issue is not institutional incapacity but the interaction between administrative law, political oversight, and fiscal risk management. This dynamic provides the empirical foundation for what this article later conceptualizes as the Capacity–Protectionism Paradox.

As synthesized in Table 1, the trajectory of institutional friction across the three jurisdictions demonstrates that sovereign resistance to international commercial arbitration is far from uniform. Rather, it develops along a discernible spectrum shaped by a state’s level of economic maturity, historical legal trajectory, and administrative capacity. The comparative matrix points to three distinct patterns of institutional strain.

**Table 1:** Macro-level comparison of institutional friction in arbitration governance.

Analytical dimensions	Cambodia	Pakistan	Thailand
Legal and institutional heritage	Civil law (transitional, post-conflict state-building)	Common law (colonial heritage, complex federal structure)	Civil law (historically sovereign, deeply)
Macroeconomic context (BRI integration)	Developing economy (high reliance on FDI for basic infrastructure and SEZs)	Lower-middle income (geopolitical anchor of the China–Pakistan economic corridor)	Upper-middle income (mature export-oriented economy, eastern economic corridor)
Domestic arbitration architecture	2006 Commercial Arbitration Law; NCAC (nascent, highly reliant on foreign models)	1940 Arbitration Act (Obsolete); Transitioning to Draft Arbitration Act 2024	Arbitration Act 2002; TAI and THAC (sophisticated, but bifurcated by Cabinet resolutions)
Primary institutional bottleneck	Absence of a dedicated Commercial Court, an underfunded bailiff system	Systemic judicial backlog; pervasive curial intervention by domestic courts	Strict bureaucratic liability laws (Tort Liability of Officials Act); political-business nexus
Sovereign defense mechanism	Institutional absence: non-enforcement driven by administrative gridlock rather than legal strategy.	Sovereign trauma: expansive interpretation of “public policy” to issue anti-arbitration injunctions.	Weaponization of law: sophisticated use of Administrative Courts to annul awards via corruption or prescription claims.

In Cambodia, the dominant friction remains largely mechanical. Driven by the imperatives of post-conflict reconstruction and a sustained reliance on foreign direct investment, the state has adopted a formally receptive stance toward international arbitration, reflected in the establishment of the National Commercial Arbitration Center (NCAC). However, the continuing absence of a specialized Commercial Court creates a significant logistical bottleneck. The resulting enforcement difficulties appear to stem less from a deliberate sovereign strategy than from the practical limitations of an underdeveloped judicial infrastructure.

Pakistan, by contrast, reflects a more volatile configuration marked by doctrinal friction and episodic judicial intervention. Operating under the geopolitical pressures associated with the China–Pakistan Economic Corridor (CPEC) and shaped by the legacy of major Investor–State Dispute Settlement (ISDS) exposure, the state has exhibited heightened sensitivity to arbitral risk. This posture is reinforced by systemic judicial backlog and continued reliance on the Arbitration Act 1940, which structurally permits extensive curial involvement. In this setting, institutional strain is closely linked to legislative inertia, whereby the persistence of an outdated statutory framework continues to invite judicial intervention rather than constrain it.

Thailand presents the most legally sophisticated paradigm, characterized by calibrated regulatory retrenchment. As an upper-middle-income economy with well-developed arbitral institutions, most notably the TAI and THAC, and a capable civil judiciary, Thailand faces few structural barriers to enforcement (Pimentel, 2016). Nevertheless, its response to large arbitral exposure has been notably strategic. Influenced by a deeply embedded political–business nexus and stringent bureaucratic liability regimes, the state has employed Cabinet resolutions and administrative law mechanisms that function as additional filters on arbitral engagement. These measures, while formally grounded in domestic law, have the practical effect of insulating public agencies from significant arbitral liability.

Taken together, the comparative evidence highlights an important paradox in contemporary transnational dispute governance: as host-state legal capacity increases, resistance to arbitral enforcement does not necessarily recede; rather, it may become more legally structured and institutionally refined. This spectrum of sovereign response provides the analytical foundation for the subsequent discussion of how advanced forms of protectionism are articulated through public policy doctrine.

### 3.4 The sovereign shielding spectrum model: conceptual logic and analytical payoff

“Sovereign shielding” describes how states use domestic legal institutions, procedural filters, and public policy reasoning to limit or delay enforcement of transnational arbitral awards (Coppack, 2026). While states have legitimate interests in protecting public order and regulatory autonomy, the problem arises when these interests become systematic shields against arbitral liability.

The model turns on three variables: institutional capacity, fiscal and political exposure, and doctrinal instrumentality. These explain why enforcement friction takes different forms across three BRI-linked jurisdictions.

Cambodia represents *capacity-driven shielding*—the low end of the spectrum. Enforcement fails not through strategy but through absence: no specialized courts, limited expertise, weak infrastructure. This is passive resistance by institutional underdevelopment.

Pakistan represents *reactive judicial shielding*—the middle. Greater legal capacity exists, but statutory obsolescence, judicial backlog, and “sovereign trauma” from high-profile investor-state disputes make courts defensive. Public policy review becomes volatile and unpredictable.

Thailand represents *strategic doctrinal shielding*—the high end. Modern arbitration laws and sophisticated courts exist, yet administrative doctrines, prescription periods, and corruption-based invalidation serve as refined instruments of resistance. This illustrates the Capacity–Protectionism Paradox: higher legal capacity makes resistance less visible and more doctrinally legitimate, while remaining equally protective.

The model’s core insight is that legal modernization alone will not solve enforcement problems. Reform must also address the incentives that lead states to weaponize public policy and administrative law against arbitral awards.

## 4 Public policy as a sovereign shield: doctrinal transformation and strategic deployment

While institutional architectures and statutory frameworks shape the procedural flow of transnational commercial disputes, the ultimate defense of sovereign liability is waged through doctrinal escalation (Wang et al., 2024). As host states confront massive arbitral awards that threaten public funds, expose domestic administrative irregularities, or generate intense political backlash, they increasingly shift their defensive posture from procedural objections to substantive legal resistance (Khoman et al., 2024). This article examines how the public policy exception is strategically deployed, analyzing its transformation from a narrow international safeguard into a powerful, malleable instrument for sovereign shielding across the jurisdictions surveyed.

### 4.1 Reinterpreting the New York convention: from safeguard to sovereign instrument

Under the 1958 Convention on the Recognition and Enforcement of Foreign Arbitral Awards (the New York Convention), international arbitral awards benefit from a robust presumption of universal enforceability (New York Convention, 1958). The overarching objective of the Convention is to facilitate global trade by ensuring that private dispute resolution is respected across borders (Born, 2021). However, Article V(2)(b) provides a vital caveat, permitting a domestic competent authority to refuse the recognition and enforcement of an award if it conflicts with the fundamental “public policy” of the forum state [International Bar Association (IBA), 2015].

The original drafters of the Convention intended this exception to be construed extremely narrowly, functioning solely as an emergency safeguard against awards that violate the most basic, universally recognized notions of morality, fundamental justice, or international *ordre public* (Campbell, 2021). However, contemporary judicial practice in emerging and mature economies reveals a profound doctrinal transformation. Because the Convention deliberately leaves the precise definition of “public policy” to the discretion of the enforcing state, domestic judiciaries have actively engaged in an interpretive broadening of the concept (Ghodoosi, 2016). Host states increasingly utilize this interpretive flexibility as a “sovereignty recalibration mechanism” (Nottage and Thanitcul, 2017). By strategically expanding the definition of public policy to encompass domestic statutory technicalities, administrative regulations, and ex post corruption narratives, states effectively deploy the exception as a sovereign shield, insulating themselves from the financial and political consequences of transnational arbitral awards (Llamzon, 2014).

### 4.2 Cambodia: institutional absence and the void of doctrinal gatekeeping

In stark contrast to the chaotic judicial interventionism in Pakistan and the sophisticated administrative shielding in Thailand discussed in the subsequent sections, Cambodia presents a fundamentally different paradigm regarding the public policy exception. In Cambodia, the non-enforcement of international arbitral awards is rarely the product of a deliberate, calculated doctrinal weaponization of Article V(2)(b) of the New York Convention [World Intellectual Property Organization (WIPO), 2006]. Instead of substantive legal resistance, foreign investors in Cambodia encounter a profound institutional absence.

Because the Cambodian legal framework currently lacks a fully operational, specialized Commercial Court, challenges to arbitral enforcement and recognition are processed by generalist judges within the traditional civil court system (Srun, 2024). These courts, still recovering from decades of post-conflict institutional fragility, often lack the deep technical expertise, specialized commercial training, and historical jurisprudence required to engage in complex, expansive interpretations of international *ordre public* (McCarthy and Un, 2017). Consequently, the Cambodian judiciary has not yet developed the capacity to engineer exact, post facto corruption narratives or to weaponize domestic statutory timelines to systematically annul unfavorable arbitral awards, as is routinely observed in mature legal systems.

Therefore, the “sovereign shield” in Cambodia is not a meticulously drafted appellate judgment nullifying an award on public policy grounds. Rather, the shield is the raw logistical friction of a transitional bureaucratic apparatus. Arbitral judgments simply languish unenforced due to administrative opacity, a lack of cohesive inter-ministerial coordination regarding state-backed mega-projects, and an under-resourced bailiff system physically incapable of executing complex transnational seizures (Herman, 2015).

By serving as the comparative baseline in this study, Cambodia empirically demonstrates a critical theoretical point: the strategic deployment of the public policy exception is intrinsically linked to a state’s legal maturity. It requires a high threshold of judicial sophistication to effectively weaponize international legal doctrines. Without this advanced legal capacity, sovereign resistance to transnational dispute resolution remains a purely mechanical failure a logistical bottleneck rather than a calculated doctrinal defense (Wang et al., 2024).

### 4.3 Pakistan: judicial intervention and doctrinal volatility

In Pakistan, the deployment of the public policy exception is characterized by pervasive judicial interventionism and doctrinal volatility, creating a climate of severe instability regarding the enforcement expectations of foreign investors (Hassan, 2012). Operating under the obsolete Arbitration Act of 1940, which actively permits sweeping judicial review, Pakistani courts have historically employed a fluid and highly expansive interpretation of public policy [World Intellectual Property Organization (WIPO), 2006]. Rather than limiting their scrutiny to fundamental violations of international justice, domestic courts have frequently invoked the public policy defense to reopen the proceedings and conduct a substantive review of the arbitral merits (*révision au fond*) (Sharif et al., 2022).

This doctrinal volatility is deeply intertwined with the “sovereign trauma” resulting from catastrophic Investor-State Dispute Settlement (ISDS) outcomes. The *Reko Diq* mining dispute, which culminated in a staggering $6 billion ICSID penalty against the state (later forcing a complex renegotiated settlement), engendered an acute defensive paranoia within the Pakistani bureaucratic and judicial apparatus (Bohmer, 2019). Consequently, in disputes involving foreign contractors, courts have demonstrated a willingness to conflate domestic legal irregularities with international public policy violations.

The protracted saga involving the *Taisei Corporation* vividly illustrates this systemic unpredictability (Jus Mundi, 2024). In such construction and infrastructure disputes, local award debtors routinely exploit the ambiguous boundaries of public policy to secure anti-arbitration injunctions or indefinitely delay execution (Saeed et al., 2025). Furthermore, as witnessed in the historical *HUBCO* jurisprudence, the Supreme Court of Pakistan has previously invalidated arbitration agreements based on allegations of fraud and corruption that were deemed inseparable from domestic public policy concerns, effectively overriding the tribunal’s jurisdiction entirely [Kluwer Arbitration Blog (Wolters Kluwer), 2025]. This continuous constitutional and judicial overlay transforms the public policy exception into an unpredictable appellate mechanism, fundamentally destabilizing the legal security required for the multi-billion-dollar projects comprising the China-Pakistan Economic Corridor (CPEC) (Abdullah and Rasool, 2026).

### 4.4 Thailand: sophisticated sovereign shielding through administrative law

While Pakistan exhibits a chaotic, interventionist approach, Thailand presents the most advanced and highly capable paradigm of doctrinal transformation. Utilizing layered legal reasoning and meticulous administrative legality reviews, the Thai state achieves a sophisticated form of sovereign shielding (Khoman et al., 2024). Unlike the logistical bottlenecks observed in Cambodia or the statutory obsolescence in Pakistan, Thailand employs a “high-capacity defensive recalibration” to systematically protect state interests against massive infrastructural arbitral losses.

This strategic deployment of public policy is vividly evident across multiple landmark commercial and treaty-based cases (Bangkok Post, 2018, 2014, 2023). In the *Hopewell* dispute concerning a multi-billion-baht concession for an elevated transit project the Supreme Administrative Court annulled the massive arbitral award rendered against the Ministry of Transport by retroactively applying a strict domestic prescription period (statute of limitations) applicable to administrative contracts (Sucharitkul, 2014). Crucially, the court engaged in an interpretive broadening of the New York Convention’s defenses, framing the domestic administrative timeline itself not merely as a procedural technicality, but as a non-derogable matter of fundamental public policy (Yaemklin, 2022).

In the equally controversial *Klong Dan* (wastewater treatment plant) case, the state successfully cultivated a highly effective corruption narrative to shield the public purse (Khoman, 2016). Following a final arbitral award ordering the government to pay the private consortium, the state utilized a domestic criminal court’s subsequent conviction of a high-ranking Pollution Control Department official for corruption as “fresh evidence” (Bangkok Post, 2022). Based on this domestic conviction, the Administrative Court set aside the finalized arbitral award, reasoning that the underlying administrative contract was fundamentally tainted by conflicts of interest and therefore violated public order and good morals (Legal 500, 2025).

This technical annulment strategy extends seamlessly to treaty-based ISDS. In the *Walter Bau* saga, following a €29 million *ad hoc* UNCITRAL award against the state regarding a tollway concession, the Thai government systematically deployed jurisdictional challenges and sovereign immunity arguments across multiple foreign forums from Switzerland to the United States and Germany for over a decade (Sucharitkul and Travaini, 2013). Thailand only facilitated enforcement of the award after the claimant escalated the matter by seizing a royal aircraft in Munich in 2011 to compel payment (Peterson, 2017).

Furthermore, in the *Kingsgate* (Chatree Gold Mine) dispute initiated under the Thailand-Australia Free Trade Agreement (TAFTA), the state demonstrated the pinnacle of parallel defensive strategies. Following the military government’s abrupt suspension of mining operations, the state launched aggressive domestic criminal and environmental investigations into alleged “corruption and other serious illegal behavior” against the foreign investor, precisely parallel to the ongoing international arbitral proceedings (Khoman et al., 2024). By layering administrative legality reviews with powerful corruption narratives and the threat of severe domestic criminal sanctions, the Thai state deploys public policy with strategic precision. This effectively recalibrates the boundaries of state sovereignty, utilizing domestic law not merely as a regulatory tool, but as a sophisticated shield to preempt damaging awards and force favorable settlements beyond the reach of transnational arbitration (Hepburn, 2019).

## 5 Discussion

### 5.1 Debating the “legal vacuum” hypothesis: a spectrum of institutional friction

Prevailing law and development scholarship has frequently attributed the failure of transnational dispute resolution in the Global South to the existence of a “legal vacuum” namely, deficiencies in adjudicative capacity, antiquated statutory frameworks, or broader institutional fragility (Trzcinski and Upham, 2014). While this explanation retains descriptive value for certain jurisdictions, the comparative evidence generated in this study reveals a more complex and differentiated pattern of resistance. Rather than uniformly diminishing with legal modernization, state resistance to arbitral enforcement appears to evolve in form and sophistication as institutional capacity matures.

The Cambodian case most closely approximates the orthodox legal vacuum model. Here, non-enforcement largely reflects structural and administrative limitations, including reliance on generalist civil courts and limited arbitral specialization. In this context, enforcement failures are better understood as mechanical or capacity-driven rather than the product of a coherent doctrinal strategy (McCarthy and Un, 2017). Cambodia therefore represents the low-capacity end of the institutional friction spectrum, where resistance is primarily passive and infrastructural.

Pakistan occupies a volatile intermediate position in which capacity deficits intersect with what this article terms “sovereign trauma.” High-profile Investor–State Dispute Settlement (ISDS) defeats notably the Reko Diq dispute have interacted with statutory obsolescence under the Arbitration Act 1940 to produce an increasingly interventionist judicial posture. Domestic courts have demonstrated a willingness to issue broad injunctions and entertain expansive public-policy objections, thereby effectively slowing or derailing arbitral proceedings (Abdullah and Rasool, 2026; Fatima, 2024). In this middle category, resistance is neither purely mechanical nor fully strategic; rather, it reflects a hybrid form of reactive legal defensiveness shaped by both institutional weakness and political sensitivity to fiscal exposure.

Thailand, however, fundamentally challenges the capacity-deficit narrative and reveals what this article conceptualizes as the Capacity–Protectionism Paradox. Despite possessing sophisticated arbitral infrastructure, including the Thai Arbitration Institute (TAI) and Thailand Arbitration Center (THAC), and a modern arbitration framework broadly aligned with UNCITRAL standards (Nottage and Thanitcul, 2017), foreign investors continue to encounter significant enforcement friction. Crucially, this friction does not stem from institutional incapacity but from regulatory overreach embedded within Thailand’s administrative law architecture.

In particular, the interaction between strict bureaucratic liability regimes such as the Tort Liability of Officials Act 1996 and the jurisdiction of the Administrative Courts has created strong incentives for risk-averse state behavior. Thai authorities possess both the legal expertise and institutional tools to mount highly sophisticated challenges to adverse awards. As illustrated by the Hopewell and Walter Bau disputes, state actors have deployed layered administrative reasoning to recharacterize contractual or procedural issues as matters of non-derogable public policy (Sucharitkul, 2014; Khoman et al., 2024). In this high-capacity setting, resistance is deliberate, legally engineered, and doctrinally refined.

Synthesizing these three trajectories, this study advances a revised theoretical proposition: legal modernization is a necessary but insufficient condition for the reliable enforcement of arbitral awards. Where political incentives to resist large fiscal liabilities remain strong, improvements in legal capacity may instead equip host states with more sophisticated doctrinal tools through which to shield sovereign interests. The empirical pattern observed across Cambodia, Pakistan, and Thailand, therefore, supports a spectrum model of institutional friction, ranging from passive capacity failure to active legal protectionism.

This reconceptualization directly challenges the linear assumptions embedded in earlier law-and-development accounts. It suggests that the key explanatory variable is not capacity alone, but the interaction between institutional maturity, fiscal exposure, and bureaucratic risk incentives. As states move up the developmental ladder, resistance to transnational dispute mechanisms does not disappear; rather, it becomes more legally complex, strategically calibrated, and politically embedded.

The spectrum model does three things: it shows enforcement failure is a continuum (not a single phenomenon), explains how resistance *evolves* rather than declines as legal systems mature, and reveals why public policy is the preferred doctrinal vehicle—simultaneously domestic, flexible, and normatively powerful enough to reframe fiscal self-protection as legal necessity.

It also refines the critique of the legal vacuum thesis. That thesis works for Cambodia (institutional weakness drives failure), only partly for Pakistan (capacity problems *plus* sovereign risk anxiety), and fails for Thailand (high capacity, yet sophisticated resistance persists). The spectrum’s core correction: the form of shielding changes with capacity, but shielding itself does not disappear.

The policy implication follows directly. Reforming statutes and adopting international arbitration templates matter, but are insufficient where political incentives reward resistance. Effective reform also requires judicial specialization, curbs on overbroad public-policy review, bureaucratic accountability, and dispute-resolution clauses designed to minimize post-award battles—hence the article’s emphasis on hybrid architectures rather than arbitration alone.

### 5.2 The “attribution asymmetry” dilemma in transnational corruption Defenses

The most significant theoretical insight emerging from this study concerns what may be described as the problem of attribution asymmetry in contemporary arbitral practice. Comparative analysis of the strategic deployment of the public policy exception, particularly in Thailand and Pakistan, reveals a structural tension within international investment jurisprudence. When faced with substantial arbitral exposure arising from large infrastructure or extractive projects, host states increasingly invoke ex post corruption narratives to invalidate underlying contracts or resist enforcement of awards. The trajectories of Thailand’s Klong Dan wastewater project and the Kingsgate mining dispute illustrate this pattern with clarity (Khoman et al., 2024).

Current arbitral jurisprudence generally reflects a strong commitment to the strict application of the clean hand’s doctrine and a corresponding zero-tolerance approach to bribery and illegality (Hwang and Lim, 2012). In principle, this stance serves important systemic goals, including the protection of transnational public policy and the deterrence of corrupt investment practices. However, when situated within the political economy of complex state–investor relationships, the rigid application of this doctrine can generate unintended distributive effects.

The comparative evidence suggests that absolute jurisdictional or admissibility dismissals in corruption-tainted cases may produce a perverse incentive structure (Teramura et al., 2024). Where tribunals decline jurisdiction entirely upon finding investor misconduct, the immediate legal consequence typically falls on the foreign investor alone. Meanwhile, the host state whose officials may have solicited, facilitated, or knowingly tolerated the corrupt conduct often escapes contractual liability and, in some instances, continues to benefit from the underlying infrastructure or investment (Reid and Zamour, 2022). This outcome creates a structural imbalance in the allocation of legal responsibility.

It is in this context that the notion of attribution asymmetry becomes analytically salient. Mature and legally sophisticated states are increasingly capable of mobilizing domestic administrative, criminal, or public law findings to reinforce corruption defenses at the enforcement stage. The Klong Dan and Kingsgate disputes demonstrate how domestic proceedings and regulatory interventions can be layered onto arbitral resistance strategies, effectively transforming bureaucratic misconduct into a potent jurisdictional defense. In such settings, the public policy exception risks functioning not merely as a safeguard of international legality but as a sovereign shield against large fiscal liabilities.

This analysis does not suggest that tribunals should dilute anti-corruption norms or tolerate investor wrongdoing. Rather, it highlights the need for a more calibrated doctrinal response that better reflects shared responsibility in corruption-affected investments. A rigid binary, either full enforcement or total dismissal, may be ill-suited to disputes involving systemic or state-linked corruption.

Accordingly, this study argues that arbitral jurisprudence should move toward a proportionality-based framework when addressing corruption defenses. Such an approach would allow tribunals to consider the relative culpability of both investor and state actors, the degree of state knowledge or participation, and the risk of sovereign unjust enrichment. Doctrinal tools such as estoppel, contributory fault, or state acquiescence may provide more equitable mechanisms for allocating consequences in complex corruption scenarios (Reid and Zamour, 2022).

Reframing the public policy exception in this manner would preserve the integrity of international anti-corruption norms while reducing the risk that host states can strategically benefit from their own institutional failures. More broadly, recognizing and addressing attribution asymmetry is essential if the international arbitration regime is to maintain credibility in an era of increasingly sophisticated state responses to adverse awards.

### 5.3 The pivot to hybridity: bypassing the public policy trap

The comparative findings developed in this study carry a broader systemic implication: traditional adversarial arbitration is increasingly entangled in domestic judicial resistance, expansive public policy review, and sophisticated illegality defenses. Recent developments in 2024–2025 reinforce both continuity and change in this pattern. In Pakistan, the Supreme Court’s decision in Taisei Corporation v. A.M. Construction Company (Pvt.) Ltd. (2024) indicates a more enforcement-supportive direction, emphasizing a narrow interpretation of Article V of the New York Convention and rejecting merits-based review of arbitral awards. This suggests a partial shift away from historically interventionist tendencies, although structural judicial sensitivities remain relevant.

At the same time, China has continued to expand its foreign-related commercial dispute resolution architecture (Zheng and Chen, 2023). Institutional developments linked to the China International Commercial Court (CICC) and related mechanisms reflect sustained efforts to professionalize cross-border dispute governance through integrated pathways for litigation, mediation, and arbitration. These mechanisms aim to reduce fragmentation in dispute resolution and provide more predictable venues for high-value commercial disputes under Belt and Road Initiative frameworks. However, these developments do not eliminate the enforcement challenges identified in this study. Instead, they highlight why hybrid mechanisms are becoming more prominent as risk-management tools. The CICC model should therefore be understood not as a replacement for arbitration, but as part of a broader procedural ecosystem designed to manage enforcement uncertainty across diverse legal systems.

Parallel to this institutional experimentation is a renewed regional interest in international commercial mediation. Mediation’s emphasis on negotiated outcomes, relational continuity, and procedural flexibility resonates strongly with long-standing commercial practices across much of Asia (Goh and Tangkiriphimarn, 2023). More importantly, mediation has gained renewed strategic significance in light of persistent enforcement frictions affecting arbitral awards.

In this context, the United Nations Convention on International Settlement Agreements Resulting from Mediation (the Singapore Convention) has emerged as a potentially important structural innovation. By enabling the direct cross-border enforcement of qualifying mediated settlement agreements, the Convention offers parties a pathway that may, in some cases, avoid the intensive public policy scrutiny associated with arbitral award enforcement under Article V(2)(b) of the New York Convention. The comparative evidence examined in this study suggests that such mechanisms may become increasingly attractive where parties anticipate elevated risks of post-award judicial intervention.

At the same time, the transformative potential of this mediation-centered shift remains uneven. The Singapore Convention has gained increasing global relevance, but its impact remains limited by uneven ratification and reservation practices across jurisdictions (Clark and Hamaiunova, 2026). Pakistan’s recent signature reflects growing openness toward mediation-based enforcement, although its reservations demonstrate continued sovereign caution in sensitive state-related disputes. China remains a signatory without full ratification, while Thailand and Cambodia have not yet joined the Convention framework. These differences reinforce the central argument of this article: even where states formally endorse transnational dispute-resolution mechanisms, they continue to preserve legal space to protect sovereignty, public assets, and regulatory autonomy. As a result, mediation-based enforcement remains an important but partial complement to arbitration rather than a complete substitute.

These developments also cast renewed light on a structural feature of the New York Convention itself. By deliberately leaving the content of “public policy” to national courts, Article V(2)(b) has long balanced uniform enforcement against sovereign regulatory autonomy. However, as comparative practice now demonstrates, this flexibility can generate significant divergence in judicial approaches. The resulting tension between minimalist and maximalist review models continues to shape enforcement outcomes across jurisdictions (Chan and Khong, 2024). A more context-sensitive or “contextual review” approach, one that distinguishes genuine violations of fundamental international norms from strategically framed administrative objections, may offer a more sustainable equilibrium.

Taken together, the evidence points toward a gradual but meaningful pivot toward hybrid and multi-modal dispute resolution frameworks in the Belt and Road context. This shift should not be understood as the displacement of arbitration, which remains central to international commercial governance. Rather, hybridity appears to be emerging as a risk-management response to increasingly sophisticated forms of sovereign resistance. Properly calibrated, integrated mechanisms that combine arbitration, mediation, and specialized transnational fora may help reduce the enforcement uncertainties documented in Cambodia, Pakistan, and Thailand.

In this evolving landscape, embracing procedural hybridity is less a matter of institutional preference than of strategic necessity. As host-state defenses become more legally refined, the resilience of transnational dispute resolution will likely depend on the ability of parties and institutions to operate across multiple, mutually reinforcing procedural pathways.

### 5.4 Operationalizing hybrid architectures as a response to sovereign shielding

Hybrid dispute-resolution architecture is a direct response to the sovereign-shielding spectrum. Because resistance can emerge at different stages and in different forms, dispute-resolution design must be equally multi-layered. A single arbitral clause is insufficient where awards must pass through weak, defensive, or protectionist domestic courts.

Three mechanisms illustrate this shift. The China International Commercial Court’s one-stop mechanism links mediation, arbitration, neutral evaluation, and court support within one framework—not to replace arbitration, but to build procedural bridges before disputes harden into enforcement crises (Supreme People’s Court of China, 2023). The Singapore Convention on mediated settlements offers a second pathway: negotiated consent reduces the adversarial escalation that typically triggers sovereign shielding. At the same time, the Convention provides cross-border enforceability without eliminating public policy review. Multi-tier dispute clauses and adjudication boards provide a third layer, requiring negotiation, technical review, or expert determination before arbitration begins—resolving disputes early enough to avoid the high-value awards that provoke public policy objections or corruption-based defenses.

Clause design should directly track the spectrum. Low-capacity jurisdictions call for procedural clarity and neutral seats. Intermediate jurisdictions require defined governing law, emergency procedures, and waiver of dilatory tactics. High-capacity but protectionist jurisdictions demand early settlement mechanisms and mediation-arbitration pathways that minimize the risk of a politically sensitive final award being subject to intensive domestic review.

Hybrid architecture is not arbitration’s failure—it is its maturation. In BRI disputes that are financially large, politically sensitive, and legally cross-border, durability depends less on arbitration’s formal availability and more on designing procedures that prevent sovereign shielding from becoming the inevitable final stage.

## 6 Conclusion

The rapid expansion of transnational capital flows under the Belt and Road Initiative (BRI) has exposed important structural tensions within the dispute-resolution architectures of several emerging Asian economies. Through a comparative examination of Thailand, Pakistan, and Cambodia, this study demonstrates that the recognition and enforcement of foreign arbitral awards is not merely a technical legal exercise. Rather, it operates within a politically sensitive space where concerns about fiscal exposure, bureaucratic liability, and sovereign control significantly shape judicial behavior.

Across the three jurisdictions, the evidence reveals multiple pathways through which enforcement may be constrained. In Thailand, the interaction between the administrative and civil court systems creates doctrinal avenues through which large arbitral liabilities can be subjected to intensive public policy scrutiny. In Pakistan, the legacy of major investor–state disputes has contributed to a historically interventionist judicial posture. However, recent jurisprudence, most notably the Supreme Court’s 2024 Taisei decision, suggests a cautious movement toward a more enforcement-supportive approach. Cambodia, by contrast, continues to face more structural and capacity-related barriers, where institutional limitations and high transaction costs still impede the routine use of formal arbitral mechanisms.

Taken together, these findings challenge the explanatory sufficiency of the traditional “legal vacuum” hypothesis and support the alternative framework of strategic sovereignty. While the legal vacuum thesis remains useful in explaining Cambodia’s capacity-driven enforcement constraints, it fails to adequately explain Pakistan’s reactive judicial defensiveness and Thailand’s sophisticated administrative-law resistance. Strategic sovereignty better captures how states actively use public policy exceptions, administrative law tools, and judicial interpretation to manage the domestic consequences of transnational arbitral awards. In lower-capacity environments, enforcement failures reflect logistical bottlenecks. In intermediate systems, they reflect defensive judicial intervention. In more legally sophisticated systems, they take the form of technically refined doctrinal resistance. Legal modernization, therefore, does not guarantee stronger enforcement; in some cases, it equips states with more sophisticated instruments of sovereign shielding.

### 6.1 Synthesizing the Spectrum of sovereign shielding

The comparative analysis supports the existence of an evolving spectrum of sovereign shielding. Cambodia largely reflects capacity-driven friction, where enforcement difficulties stem from institutional underdevelopment and limited specialization. Pakistan occupies an intermediate position, characterized by doctrinal volatility, in which statutory legacy issues and prior ISDS exposure have encouraged expansive judicial intervention. Thailand presents the most theoretically significant case. Despite possessing modern arbitration legislation and reputable arbitral institutions, the Thai legal system demonstrates how sophisticated administrative and public law tools can be mobilized to scrutinize high-value awards. The Thai experience illustrates what this study has termed the Capacity–Protectionism Paradox: enhanced legal capacity may, under certain political and institutional conditions, enable more refined forms of resistance rather than the disappearance of resistance.

### 6.2 Resolving the dilemma of attribution asymmetry

A second major contribution of this research concerns the identification of attribution asymmetry in corruption-affected disputes. The case studies suggest that in some high-stakes infrastructure conflicts, corruption allegations may emerge late in the dispute cycle and become intertwined with enforcement-resistance strategies. When arbitral tribunals apply the clean hands doctrine in an uncompromising binary fashion, the legal consequences may fall disproportionately on investors, even when state officials were materially involved in the underlying misconduct.

This study does not question the central importance of robust anti-corruption norms in international arbitration. However, it argues that the current jurisprudential trajectory risks producing uneven accountability in cases involving systemic or state-linked corruption. To preserve both legitimacy and deterrence, tribunals may need to adopt more calibrated approaches that account for relative fault, state knowledge, and the risk of unjust enrichment by sovereigns. Doctrinal tools such as proportionality analysis, estoppel, or contributory responsibility warrant further development in this context.

### 6.3 Strategic implications for transnational dispute governance

The central lesson is that enforcement risk turns not on whether a state has modern arbitration law, but on where it sits on the sovereign shielding spectrum—logistical non-enforcement at the low-capacity end, reactive judicial intervention in the middle, and refined doctrinal resistance at the high-capacity end.

First, greater doctrinal clarity around public policy is necessary. Article V(2)(b) of the New York Convention legitimately preserves national autonomy, but an overbroad interpretation turns public policy into a general appellate device. Courts should distinguish genuine violations of fundamental public order from strategic reliance on administrative technicalities.

Second, domestic reform must go beyond legislation. Pakistan needs judicial training and limits on curial intervention alongside statutory updates. Thailand shows that modern laws are insufficient where administrative structures incentivize defensive resistance. Cambodia still requires basic institutional capacity. Each jurisdiction needs a reform strategy matched to its position on the spectrum.

Third, hybrid architectures should be treated as a practical response to enforcement fragility. Multi-tier clauses, dispute boards, the CICC, and mediation mechanisms reduce the likelihood that disputes escalate into politically sensitive awards that trigger shielding—complementing arbitration rather than replacing it.

Fourth, the Singapore Convention offers a valuable but partial supplement. Mediated settlements preserve relationships and reduce political costs, but uneven ratification and implementation limit their current reach (Thongkliangket, 2022).

Finally, investors and state entities should incorporate the sovereign shielding spectrum into contract design. Cambodia-like environments call for procedural clarity and neutral seats. Pakistan-like environments require unambiguous governing law and enforcement procedures. Thailand-like environments demand attention to administrative compliance, limitation periods, anti-corruption safeguards, and early resolution mechanisms.

### 6.4 Power asymmetry and strategic sovereignty in Chinese-led legal integration

The findings must also be situated within broader power asymmetries embedded in Chinese-led economic integration. These asymmetries reflect differences in economic scale, institutional capacity, and dependence on external capital and infrastructure financing. Such conditions shape how legal norms are received and contested in host states. However, the evidence from Cambodia, Pakistan, and Thailand shows that host states are not passive recipients of external legal influence. Instead, they exercise different forms of agency in shaping dispute resolution outcomes. Institutional limitations constrain Cambodia’s agency; Pakistan demonstrates reactive legal defensiveness shaped by fiscal and sovereign risk concerns; Thailand exercises more sophisticated administrative and doctrinal control through public policy and administrative law mechanisms.

Accordingly, the distinction between “legal vacuum” and “strategic sovereignty” is essential. The legal vacuum thesis explains capacity deficits but cannot account for strategic legal resistance in more developed systems. Strategic sovereignty better explains how legal doctrines, bureaucratic structures, and judicial interpretation are used to manage the distributive consequences of transnational economic integration.

### 6.5 Future research directions

This study also highlights several avenues for further research. Empirical work is needed to assess the real-world enforcement performance of emerging hybrid fora, including the CICC, across different Asian jurisdictions. Longitudinal studies examining how courts interpret and apply the Singapore Convention, particularly in disputes involving state-owned entities, would also be valuable. Finally, further doctrinal analysis is required to refine the operational boundaries of estoppel, proportionality, and related doctrines in corruption-affected arbitration.

## Data Availability

The original contributions presented in the study are included in the article/supplementary material, further inquiries can be directed to the corresponding author.
